# METTL9-catalyzed histidine methylation of S100A9 suppresses the anti-*Staphylococcus aureus* activity of neutrophils

**DOI:** 10.1093/procel/pwad047

**Published:** 2023-07-31

**Authors:** Dan Cao, Mengyue Lv, Chi Hu, Shukai Li, Siwen Wang, Chao Xu, Wen Pan

**Affiliations:** Department of Digestive Disease, The First Affiliated Hospital of USTC, Division of Life Sciences and Medicine, University of Science and Technology of China, Hefei 230001, China; Division of Life Sciences and Medicine, The CAS Key Laboratory of Innate Immunity and Chronic Disease, School of Basic Medical Sciences, Institute of Immunology, University of Science and Technology of China, Hefei 230027, China; Department of Digestive Disease, The First Affiliated Hospital of USTC, Division of Life Sciences and Medicine, University of Science and Technology of China, Hefei 230001, China; Division of Life Sciences and Medicine, The CAS Key Laboratory of Innate Immunity and Chronic Disease, School of Basic Medical Sciences, Institute of Immunology, University of Science and Technology of China, Hefei 230027, China; Department of Digestive Disease, The First Affiliated Hospital of USTC, Division of Life Sciences and Medicine, University of Science and Technology of China, Hefei 230001, China; Division of Life Sciences and Medicine, The CAS Key Laboratory of Innate Immunity and Chronic Disease, School of Basic Medical Sciences, Institute of Immunology, University of Science and Technology of China, Hefei 230027, China; Department of Digestive Disease, The First Affiliated Hospital of USTC, Division of Life Sciences and Medicine, University of Science and Technology of China, Hefei 230001, China; Division of Life Sciences and Medicine, The CAS Key Laboratory of Innate Immunity and Chronic Disease, School of Basic Medical Sciences, Institute of Immunology, University of Science and Technology of China, Hefei 230027, China; Department of Digestive Disease, The First Affiliated Hospital of USTC, Division of Life Sciences and Medicine, University of Science and Technology of China, Hefei 230001, China; Division of Life Sciences and Medicine, The CAS Key Laboratory of Innate Immunity and Chronic Disease, School of Basic Medical Sciences, Institute of Immunology, University of Science and Technology of China, Hefei 230027, China; Division of Life Sciences and Medicine, MOE Key Laboratory for Cellular Dynamics, Hefei National Center for Cross-Disciplinary Sciences, University of Science and Technology of China, Hefei 230027, China; Department of Digestive Disease, The First Affiliated Hospital of USTC, Division of Life Sciences and Medicine, University of Science and Technology of China, Hefei 230001, China; Division of Life Sciences and Medicine, The CAS Key Laboratory of Innate Immunity and Chronic Disease, School of Basic Medical Sciences, Institute of Immunology, University of Science and Technology of China, Hefei 230027, China

## Abstract

**Graphical Abstract:**
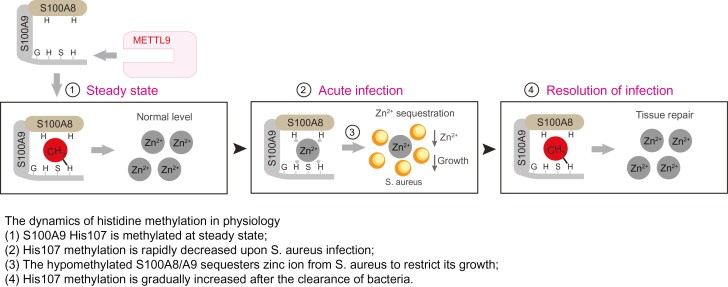

## Dear Editor,

Protein histidine methylation has been found to be a widespread post-translational modification (PTM) in mammalian cells and represents more than 13% of all protein methylation events in the human methylome (Ning et al., 2016; Kapell and Jakobsson, 2021). Recently, we and other groups independently identified METTL9 as the first broad-specificity histidine N1 methyltransferase in mammals (Davydova et al., 2021; Lv et al., 2021). METTL9 methylates a variety of substrates with a common x-His-x-His motif (His is for histidine; the second x is preferably small amino acids: A/C/G/S) and catalyzes methyl transfer to the last histidine. Although METTL9-mediated histidine N1 methylation is pervasive in mammalian cells, its pathophysiological functions are undescribed. None of the *in vivo* phenotypes of *Mettl9*^−/−^ mice have been reported thus far (Kwiatkowski and Drozak, 2020; Davydova et al., 2021; Jakobsson, 2021).

Alarmin S100A9, which heterodimerizes with S100A8, is constitutively expressed in neutrophils and monocytes. Once released upon infection, the S100A8/A9 complex exhibits broad-spectrum antimicrobial activity against numerous microorganisms (Roth et al., 2001) by sequestering essential trace metals such as Zn^2+^ and Mn^2+^, which are required for bacterial growth (Wang et al., 2018). S100A9 was found to carry histidine methylation at the His107 residue in previous mass spectrum data (Raftery et al., 1996). We and other groups have previously reported that METTL9 methylates S100A9 at the His107 residue (Daitoku et al., 2021; Davydova et al., 2021; Lv et al., 2021). However, whether METTL9-catalyzed His107 methylation of S100A9 has bona fide physiological functions remains unknown. Consistent with previous works, we confirmed by *in vitro* methylation assays that METTL9 methylates S100A9 at residue His107; no methylation was detected upon mutating S100A9’s His107 residue to Gly (Fig. S1A).

**Figure 1.**
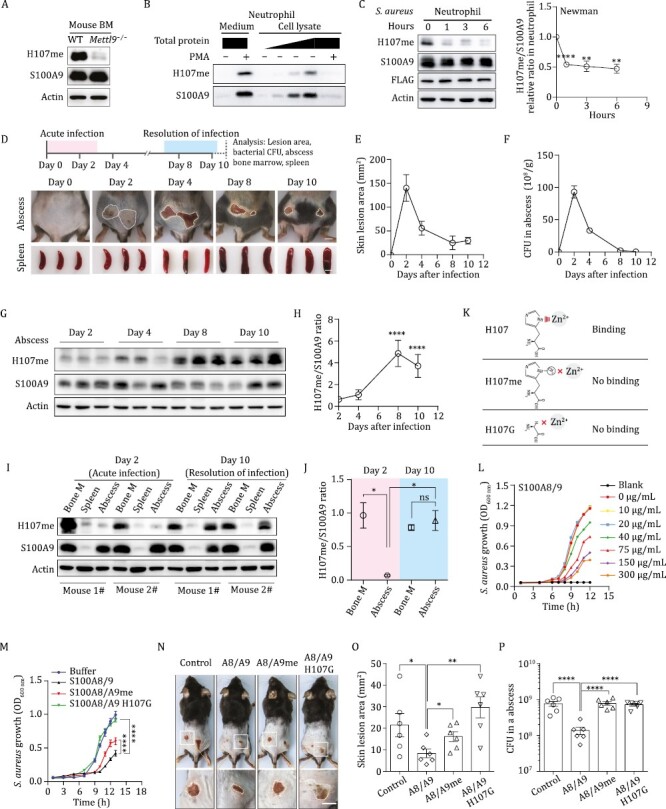
**His107 methylation of S100A9 is reduced at the acute stage of *S. aureus* infection, and His107 methylation reduces the anti-*S. aureus* function of S100A8/A9.** (A) Immunoblotting analysis of S100A9 His107me in bone marrow (BM) cells from WT and *Mettl9*^−/−^ mice. (B) Purified mouse primary neutrophils were treated with 100 nmol/L PMA for 4 h. Cell lysates were extracted from cells, and supernatants (medium) were concentrated with Millipore® devices (cut-off 3 kDa), followed by immunoblotting analysis of S100A9 His107me levels. The loading of total proteins is indicated. (C) Representative immunoblotting analysis of His107me and total S100A9 protein in mouse peritoneal neutrophils stimulated with *S. aureus* (MOI 1:10, 10 bacteria per neutrophil) for the indicated period of time. Error bars represent SEM (D–F) WT mice were subcutaneously inoculated with 3 × 10^8^*S. aureus*, and the mice were euthanized every 2 or 4 days after infection. Scale bar, 5 mm. (D) Top: the workflow of analysis of *S. aureus* infection on the indicated day. Bottom: representative images of skin lesions and spleen sizes on the indicated days. (E) Statistical plots of skin lesion area from mice in (D). (F) Quantification of bacterial CFUs in infected skin abscesses in (D). Error bars represent SEM (G) Representative immunoblotting analysis of His107me and total S100A9 protein in abscess tissue of infected mice in (D). (H) The relative ratio of methylated S100A9 to total S100A9 in (G) was calculated. Error bars represent S.D. (I) Representative immunoblotting analysis of His107me and total S100A9 protein in BM, spleen and abscess tissue of infected mice in (D) at day 2 and day 10. (J) The ratio of methylated S100A9 to total S100A9 in (I) was calculated. Error bars represent S.D. (K) Schematic of WT histidine, methylated histidine, and glycine, as well as their binding mode with zinc. (L and M) The *in vitro* anti-*S. aureus* assays of the S100A8/A9 recombinant proteins. The optical density measurements of *S. aureus* growth at a wavelength of 600 nm (OD_600 nm_) (L) in the presence of unmethylated S100A8/A9 proteins at the indicated concentrations; (M) in the presence of unmethylated S100A8/A9, methylated S100A8/A9 or H107G mutated S100A8/A9. Error bars represent S.D. (N–P) WT mice were subcutaneously inoculated with a mixture of 2 × 10^8^*S. aureus* and 30 µg of the indicated recombinant proteins per mouse. (N) Representative images of skin lesions from mice in different groups. Scale bar, 5 mm. (O) Statistical plots of skin lesion areas from mice in (N) at day 3 after infection. Each dot represents one lesion. *n* = 6. Error bars represent SEM (P) Bacterial CFUs were quantified in the infected skin abscess tissue isolated from mice in (N). Error bars represent SEM For all panels, *P < 0.05; **P < 0.01; ****P < 0.0001; ns: not significant. Data for all panels are representative of two or more independent experiments.

To better detect His107 methylation of S100A9 *in vivo*, we generated site-specific polyclonal antibodies to detect His107 methylation. We tested the specificity of these antibodies by incubating recombinant GST-tagged S100A9 with or without recombinant METTL9 in the presence of *S*-adenosylmethionine (SAM), followed by immunoblotting with these antibodies. Methylation of recombinant GST-tagged S100A9 was detected only in the presence of METTL9, suggesting the good quality of our antibodies (Fig. S1B). To confirm that METTL9 methylates S100A9 *in vivo*, we generated a *Mettl9*-deficient (*Mettl9*^−/−^) mouse strain [see Fig. S1C–E for the gene knockout (KO) strategy and confirmation of gene knockout expression] and further harvested WT and *Mettl9*^−/−^ bone marrow (BM) cells. Immunoblotting showed that in WT cells that expressed METTL9, S100A9 His107 was methylated; in contrast, almost no His107 methylation was detected in *Mettl9*^−/−^ cells (Fig. 1A). We next assessed the methylation status of secreted S100A9, as S100A9 is secreted upon neutrophil activation (Wang et al., 2018). Immunoblotting of the culture medium samples from PMA-stimulated mouse primary neutrophils showed that secreted S100A9 can also be methylated (Fig. 1B).

Using site-specific antibodies, we evaluated whether His107 methylation of S100A9 was relatively static or dynamic during bacterial infection. Due to our failure to obtain effective METTL9 antibodies, we engineered a *Mettl9* FLAG knock-in (KI) mouse strain wherein a FLAG tag was inserted into the endogenous *Mettl9* locus (see Fig. S2A–C for the gene KI strategy and confirmation of gene KI expression). We next isolated peritoneal neutrophils from *Mettl9* FLAG KI mice and treated the cells with *S. aureus* for one to 6 h, followed by immunoblotting with the indicated antibodies. Although the protein levels of FLAG-fused METTL9 remained similar, the His107 methylation of S100A9 was rapidly reduced in neutrophils upon *S. aureus* infection (Fig. 1C).

To next assess whether His107 methylation of S100A9 is also reduced *in vivo* during bacterial infection, we utilized a murine model of *S. aureus* skin infection, in which neutrophils play a primary role in the innate immune response that controls *S. aureus* infection by forming an abscess to wall off the infection and facilitate bacterial clearance (Kobayashi et al., 2015). Specifically, we induced *S. aureus* skin infection in WT mice, euthanized the animals at different time points after infection, and monitored the disease states by measuring the lesion area, spleen size, local bacterial CFUs, and recruitment of innate immune cells into infected sites (Fig. 1D). The subcutaneous injection of *S*. *aureus* resulted in the formation of a measurable lesion starting on days 1–2, with a maximum size achieved on ~day 2, followed by resolution of infection and a decrease in the lesion area (Fig. 1E). The bacterial burden in the abscess was high at the acute stage (days 0–2) and then gradually decreased to a low level at the resolving stage (days 8–10) (Fig. 1F). Consistently, neutrophils/monocytes were rapidly recruited into infected sites after onset and maintained a level of approximately 95% of total immune cells throughout the infection (Fig. S3A–C).

To examine the protein levels of His107 methylated and total S100A9, we harvested local abscess-infiltrating cells, BM cells and spleen cells at different time points during *S*. *aureus* infection. Given that it was not feasible to obtain abscess tissue at day 0, we mainly evaluated His107 methylation of S100A9 at the acute stage (day 2) and the resolving stage (days 8–10) of infection. In abscess-infiltrating cells, His107 methylation was significantly reduced (~5-fold) at day 2 compared with days 8**–**10 (Fig. 1G and 1H). Consistently, His107 methylation in abscesses was significantly lower than that in BM at day 2, when the bacterial CFU was high, while His107 methylation in abscesses increased by ~4-fold and exhibited no appreciable difference from that in BM at day 10, when most of the bacteria had been cleared (Fig. 1I and 1J). Together, these data suggest that His107 methylation of S100A9 is significantly decreased at local infected sites during the acute stage of *S. aureus* infection.

We next asked how His107 methylation affects the antibacterial activity of S100A9. Human S100A9 His105 residue, as well as mouse His107 residue, are structurally characterized as a zinc or manganese-binding site (Damo et al., 2013). From structural perspectives, His107 methylation, as well as its H107G mutation, would both lead to decreased zinc or manganese-binding compared to His107 (Fig. 1K). Indeed, His107 methylation of an S100A9 peptide (16 amino acids) showed reduced zinc-binding activity (Daitoku et al., 2021).

To examine whether a recombinant full-length methylated S100A9 (S100A9me) protein had reduced zinc-binding, we co-transformed the *S100a9* plasmid with and without the *Mettl9* plasmid into *Escherichia coli* bacteria and purified the S100A9 protein (see Fig. S4A–C and Methods for detailed protocols of protein purification). Immunoblotting and mass spectrometry showed that the purified S100A9 protein was methylated at the His107 residue when the *S100a9* plasmid was co-transformed with the *Mettl9* plasmid; no His107 methylation was detected when the *S100a9* plasmid was transformed alone (Fig. S4B and S4C). Using an ITC-binding assay, we confirmed that full-length methylated S100A9 had significantly reduced zinc-binding activity compared to its unmethylated control (*K*_*d*_s: 90.9 µmol/L vs. 14.3 µmol/L) (Fig. S4D).

Given that the zinc-binding site is important for the antimicrobial function of S100A8/A9 (Corbin et al., 2008), we next assessed whether His107 methylation affects the anti-*S. aureus* activity of S100A8/A9 *in vitro*. Using similar methods as above, we purified S100A8/A9 with and without His107 methylation (Fig. S4E). We also constructed an *S100a9* mutant plasmid with its His107 residue mutated to Gly (H107G) and purified the recombinant S100A8/A9 H107G complex. Next, we incubated the recombinant S100A8/A9 protein with *S. aureus* and monitored the growth of *S. aureus* by measuring the optical density (OD_600 nm_) (see Methods for a detailed protocol). As a positive control, unmethylated S100A8/A9 protein exhibited effective anti-*S. aureus* activities in a dose-dependent manner (Fig. 1L), indicating that our purified S100A8/A9 protein was functional and that the *in vitro* anti-*S. aureus* assay was successful. Using this assay, we found that both methylated S100A8/A9 and H107G-mutated S100A8/A9 significantly reduced their anti-*S. aureus* functions compared to unmethylated S100A8/A9 (Fig. 1M).

We further explored whether His107 methylation reduces the anti-*S. aureus* function of S100A8/A9 *in vivo*. Specifically, we subcutaneously administered 30 µg of different forms of recombinant S100A8/A9 proteins, together with 2 × 10^8^*S. aureus,* on the backs of WT mice and monitored the skin lesion area daily. We observed that WT mice administered unmethylated S100A8/A9 had significantly reduced local lesion areas and bacterial CFU loads in the abscess tissue compared to those of WT mice administered methylated S100A8/A9 and H107G mutated S100A8/A9 (Fig. 1N–P), indicating that His107 methylation indeed reduces anti-*S. aureus* function of S100A8/A9 *in vivo*.

To further investigate the physiological function of METTL9-catalyzed His107 methylation of S100A9 *in vivo*, we took advantage of *Mettl9*^−/−^ mice, in which His107 methylation of S100A9 was completely blocked (Fig. 1A). At steady state, *Mettl9*^−/−^ mice were healthy and had no obvious weight abnormalities. Six- to eight-week-old WT and *Mettl9*^−/−^ mice had similar numbers and percentages of major cell populations, including neutrophils, monocytes, and macrophages, in their BM (Fig. S1F and S1G).

**Figure 2.**
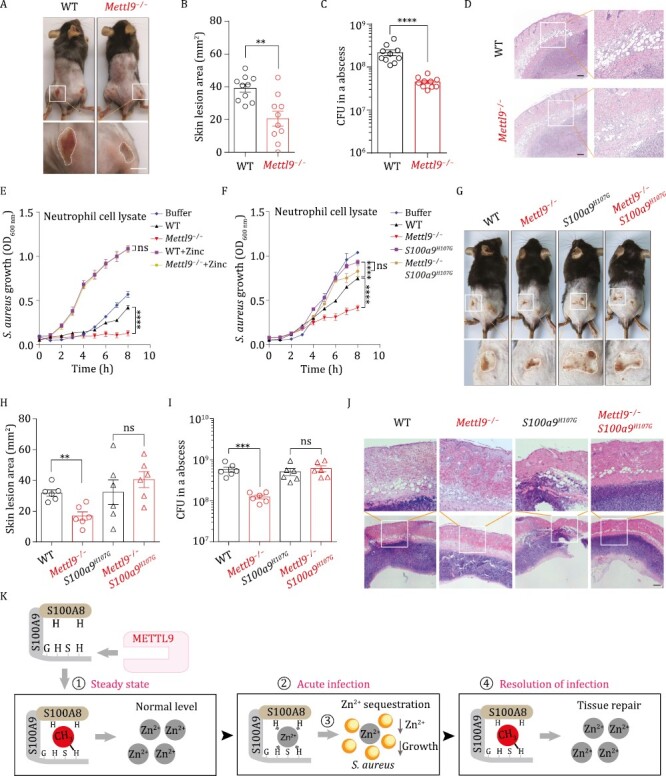
***S100a9*** ^***H107G***^ **mutation abolishes the increased anti-*S. aureus* activity observed upon deletion of *Mettl9*.** (A–D) WT or *Mettl9*^*−/−*^ mice were subcutaneously inoculated with 3 × 10^8^*S. aureus*. (A) Representative images of skin lesions from WT or *Mettl9*^*−/−*^ mice. Scale bar, 5 mm. (B) Statistical plots of skin lesion areas from WT or *Mettl9*^*−/−*^ mice at day 5 after infection. Each dot represents one lesion. *n* = 10. Error bars represent SEM (C) Bacterial CFUs were quantified in the infected skin abscess tissue isolated from mice in (B). Error bars represent SEM (D) Representative H&E staining images of skin abscess tissue from infected WT or *Mettl9*^*−/−*^ mice. Scale bar, 200 µm. (E) Analysis of *S. aureus* growth in the presence of WT or *Mettl9*^*−/−*^ neutrophil lysates (350 µg/mL) with 100 µmol/L TPEN or with 100 µmol/L TPEN plus 500 mmol/L zinc in TSB medium. Error bars represent S.D. (F) Analysis of *S. aureus* growth in the presence of WT, *Mettl9*^*−/−*^, *S100a9*^*H107G*^, and *Mettl9*^*−/−*^*S100a9*^*H107G*^ neutrophil lysates (500 µg/mL) with 40 µmol/L TPEN in TSB medium. Error bars represent S.D. (G–I) WT, *Mettl9*^−/−^, *S100a9*^*H107G*^ and *Mettl9*^−/−^*S100a9*^*H107G*^ mice were subcutaneously inoculated with 3 × 10^8^*S. aureus*. (G) Representative images of skin lesions from mice in different groups. Scale bar, 5 mm. (H) Statistical plots of skin lesion areas from mice in (G) at day 3 after infection. Each dot represents one lesion. *n* = 6. Error bars represent SEM (I) Bacterial CFUs were quantified in the infected skin abscess tissue isolated from mice in (G). Error bars represent SEM (J) Representative H&E staining images of skin abscess tissue from infected mice in (G). Scale bar, 200 µm. For all panels, **P < 0.01; ***P < 0.001; ****P < 0.0001; ns: not significant. Data for all panels are representative of two or more independent experiments. (K) The working model for the METTL9-S100A9 His107me axis in the regulation of the host’s anti-*S. aureus* response: (i) S100A9 His107 is methylated at steady state; (ii) His107 methylation of S100A9 is rapidly decreased upon *S. aureus* infection; (iii) Hypomethylated S100A8/A9 sequesters zinc ions from *S. aureus* to restrict its growth; and (iv) His107 methylation of S100A9 is gradually increased after the clearance of bacteria.

Given that His107 methylation reduces anti-*S. aureus* function of S100A8/A9, we postulated that *Mettl9* KO mice might have increased antibacterial activity against *S. aureus* infection. Specifically, we inoculated WT and *Mettl9*^−/−^ mice subcutaneously on the back with 3 × 10^8^*S. aureus*, monitored the disease state daily by measuring the skin lesion area, and euthanized the mice 5 days after infection. Compared to WT control mice, *Mettl9*^−/−^ mice exhibited a significantly decreased lesion area and *S. aureus* burden in the abscess tissue (Fig. 2A–C). H&E staining of abscess tissue showed that *Mettl9*^−/−^ mice had reduced skin thickness and local inflammation (Fig. 2D). These data suggested that *Mettl9*^−/−^ mice indeed had an increased anti-*S. aureus* phenotype. Although the recruitment of neutrophils in abscesses showed no significant differences between WT and *Mettl9*^−/−^ mice (Fig. S5A), the antimicrobial activity of neutrophils might increase in *Mettl9*^−/−^ mice.

Neutrophils use multiple antimicrobial mechanisms when engaging *S. aureus*. In addition to S100A8/A9-mediated metal chelation (Corbin et al., 2008), neutrophils can phagocytose microbes and are activated to produce reactive oxygen species (ROS) (DeLeo et al., 1999). Neutrophils can also release neutrophil extracellular traps (NETs) to kill microbes (DeLeo and Allen, 2020). We observed that upon activation with phorbol 12-myristate 13-acetate (PMA), WT and *Mettl9*^−/−^ neutrophils induced similar levels of ROS (Fig. S5B) and NETs (Fig. S5C and S5D), suggesting that these two mechanisms might not be the major contributors to the anti-*S. aureus* phenotype observed in *Mettl9*^−/−^ mice.

We next evaluated whether neutrophil lysates from *Mettl9*^−/−^ mice have increased anti-*S. aureus* activity. Previous studies demonstrated that S100A8/A9 does not contribute to neutrophil phagocytic killing, but neutrophil cytoplasmic extracts containing S100A8/A9 inhibit bacterial growth through metal chelation (Roth et al., 2001; Corbin et al., 2008). Briefly, we extracted cell lysates from peritoneal neutrophils, incubated the lysates with *S. aureus* in TSB medium, and monitored bacterial growth by measuring the optical density (OD_600 nm_) for 8 h. Since TSB medium is rich in zinc, we serially diluted the zinc chelator *N*,*N*,*Nʹ*,*Nʹ*-tetrakis (2-pyridylmethyl) ethylenediamine (TPEN) to set up a low-zinc culture condition wherein *S. aureus* still grew normally (Fig. S6A). Under the optimal TPEN concentration, as the amounts of neutrophil lysates increased, their inhibition of *S. aureus* growth increased and exhibited a dose-dependent effect (Fig. S6B–I), indicating that the lysate-based anti-*S. aureus* assay was successful. Using this assay, we determined that the lysates from *Mettl9*^−/−^ neutrophils indeed exhibited significantly increased anti-*S. aureus* ability compared to that from WT neutrophils, while adding excessive zinc into the lysates led to a rescue of anti-*S. aureus* activity (Fig. 2E), confirming that *Mettl9*^−/−^ neutrophil lysates increase the anti-*S. aureus* activity in a zinc-dependent antibacterial mechanism.

We next sought to determine whether the increased anti-*S. aureus* phenotype in *Mettl9*^−/−^ mice was attributed to the loss of S100A9 His107 methylation. We reasoned that if the His107 residue was functionally important for the increased antibacterial activity of *Mettl9*^−/−^ mice, we would expect that mutation of this residue could lead to a rescue of antibacterial activity. We tested this possibility by producing an *S100a9*^*H107G*^ point-mutated mouse strain (see Fig. S7A–C for the CRISPR/Cas9 point-mutation KI strategy and confirmation of point-mutation expression) and further obtained a *Mettl9*^−/−^*S100a9*^*H107G*^ double-deficient mouse strain.

We first assessed the anti-*S. aureus* activity of neutrophil lysates from WT, *Mettl9*^−/−^, *S100a9*^*H107G*^ and *Mettl9*^−/−^*S100a9*^*H107G*^ mice. Similar to the aforementioned phenotypes, the lysates from *Mettl9*^−/−^ neutrophils exhibited significantly increased anti-*S. aureus* ability compared to that of WT neutrophils. Lysates from *Mettl9*^−/−^*S100a9*^*H107G*^ mice showed no appreciable difference from those from *S100a9*^*H107G*^ mice, suggesting that mutation of the His107 residue led to a significant rescue of the antibacterial activity observed in *Mettl9*^−/−^ neutrophil lysates (Fig. 2F).

We further assessed the anti-*S. aureus* phenotype of WT, *Mettl9*^−/−^, *S100a9*^*H107G*^ and *Mettl9*^−/−^*S100a9*^*H107G*^ mice *in vivo*. Specifically, we inoculated these mice subcutaneously on the back with 3 × 10^8^*S. aureus*, monitored the skin lesions daily and euthanized the mice three days after infection. Similarly, *Mettl9*^−/−^ mice exhibited a significantly decreased lesion area and *S. aureus* burden in the abscess tissue compared to WT controls (Fig. 2G). In contrast, no significant changes were observed when comparing the lesion sizes and CFU load in *Mettl9*^−/−^*S100a9*^*H107G*^ mice to those in *S100a9*^*H107G*^ mice (Fig. 2H–J). Notably, although *S100a9*^*H107G*^ neutrophil lysates had a significantly increased anti*-S. aureus* activity compared to WT lysates *in vitro* (Fig. 2F), *S100a9*^*H107G*^ mice did not show an obvious antimicrobial phenotype compared to WT mice (refer to the Discussion). Nevertheless, these data demonstrate that the *S100a9*^*H107G*^ mutation largely abolished the increased anti-*S. aureus* function observed upon deletion of *Mettl9*, suggesting that METTL9 controls *S. aureus* infection via the S100A9 His107 axis *in vivo*.

In the PTM field, the dynamic and spatiotemporal study of protein PTMs is important to fully understand their complex roles in physiology and disease. As a rarely studied modification, our data reveal that protein histidine methylation could be dynamic (Fig. 2K): (i) The METTL9-S100A9 His107 methylation axis suppresses the antibacterial activity of S100A8/A9 at steady state; (ii) Upon *S. aureus* infection, the ratio of methylated/total S100A9 is markedly reduced at the acute stage, which endows S100A8/A9 with increased zinc-binding and zinc-dependent anti-*S. aureus* activity; (iii) The ratio is gradually increased at the resolving stage to act as a brake on the host’s anti-*S. aureus* activity.

In summary, our work reports the profound physiological significance of the METTL9-S100A9 His107me axis in the regulation of the host’s antibacterial responses and deepens our current understanding of the chemical, enzymological, and physiological aspects of protein histidine methylation beyond protein lysine/arginine methylation. This work will open up new perspectives for future research on the importance of histidine methylation of proteins in eukaryotes.

## Supplementary Material

pwad047_suppl_Supplementary_Materials

## Footnotes

We thank the proteomics platform and imaging platform of the core facility center for life sciences, USTC. We thank the platform of autoradiography in the core facility center for life sciences. We thank Dr. Baolin Sun at USTC for the gift of S. aureus Newman. We thank the assistance from Dr. Xuebiao Yao’s laboratory at USTC. We thank Dr. Shu Zhu at USTC for useful discussion.

This work was supported by NSFC Grant No. 81872327 (to WP), “USTC Important Direction” Cultivation Project (WK3520000013) (to WP), and “The Fundamental Research Funds for the Central Universities” (WK9110000065) (to DC). “The Fundamental Research Funds of USTC” (2019ZC027) (to DC).

The authors declare no competing interests. All institutional and national guidelines for the care and use of laboratory animals were followed. The datasets generated during and/or analysed during the current study are available from the corresponding author on reasonable request.

DC, ML and WP designed the experiments; DC and ML performed the experiments for S100A8/A9 and *in vivo* immunology experiments; CH collected all MS data. SW and SL participated in the experiments. CX provided critical comments and suggestions. WP supervised the project. DC and WP wrote the manuscript with input from others.

## References

[CIT0001] Corbin BD , Seeley EH, Raab A et al. Metal chelation and inhibition of bacterial growth in tissue abscesses. Science 2008;319:962–965. 18276893 10.1126/science.1152449

[CIT0002] Daitoku H , Someya M, Kako K et al. siRNA screening identifies METTL9 as a histidine Nπ-methyltransferase that targets the proinflammatory protein S100A9. J Biol Chem 2021;297:101230. 34562450 10.1016/j.jbc.2021.101230 PMC8571522

[CIT0003] Damo SM , Kehl-Fie TE, Sugitani N et al. Molecular basis for manganese sequestration by calprotectin and roles in the innate immune response to invading bacterial pathogens. Proc Natl Acad Sci USA 2013;110:3841–3846. 23431180 10.1073/pnas.1220341110 PMC3593839

[CIT0004] Davydova E , Shimazu T, Schuhmacher MK et al. The methyltransferase METTL9 mediates pervasive 1-methylhistidine modification in mammalian proteomes. Nat Commun 2021;12:891. 33563959 10.1038/s41467-020-20670-7 PMC7873184

[CIT0005] DeLeo FR , Allen LH. Phagocytosis and neutrophil extracellular traps. Fac Rev 2020;9:25. 33659957 10.12703/r/9-25 PMC7886055

[CIT0006] DeLeo FR , Allen LA, Apicella M et al. NADPH oxidase activation and assembly during phagocytosis. J Immunol 1999;163:6732–6740. 10586071

[CIT0007] Jakobsson ME. Enzymology and significance of protein histidine methylation. J Biol Chem 2021;297:101130. 34461099 10.1016/j.jbc.2021.101130 PMC8446795

[CIT0008] Kapell S , Jakobsson ME. Large-scale identification of protein histidine methylation in human cells. NAR Genom Bioinform 2021;3:lqab045. 34046594 10.1093/nargab/lqab045 PMC8140740

[CIT0009] Kobayashi SD , Malachowa N, DeLeo FR. Pathogenesis of *Staphylococcus aureus* abscesses. Am J Pathol 2015;185:1518–1527. 25749135 10.1016/j.ajpath.2014.11.030 PMC4450319

[CIT0010] Kwiatkowski S , Drozak J. Protein histidine methylation. Curr Protein Pept Sci 2020;21:675–689. 32188384 10.2174/1389203721666200318161330

[CIT0011] Lv MY , Cao D, Zhang L et al. METTL9 mediated N1-histidine methylation of zinc transporters is required for tumor growth. Protein Cell 2021;12:965–970. 34218407 10.1007/s13238-021-00857-4 PMC8674392

[CIT0012] Ning Z , Star AT, Mierzwa A et al. A charge-suppressing strategy for probing protein methylation. Chem Commun 2016;52:5474–5477. 10.1039/c6cc00814c 27021271

[CIT0013] Raftery MJ , Harrison CA, Alewood P et al. Isolation of the murine S100 protein MRP14 (14 kDa migration-inhibitory-factor-related protein) from activated spleen cells: Characterization of post-translational modifications and zinc binding. Biochem J 1996;316:285–293. 8645219 10.1042/bj3160285 PMC1217336

[CIT0014] Roth J , Goebeler M, Sorg C. S100A8 and S100A9 in inflammatory diseases. Lancet 2001;357:1041. 10.1016/S0140-6736(05)71610-X 11293617

[CIT0015] Wang S , Song R, Wang Z et al. S100A8/A9 in inflammation. Front Immunol 2018;9:1298. 29942307 10.3389/fimmu.2018.01298 PMC6004386

