# Supplementary material for: METTL9-catalyzed histidine methylation of S100A9 suppresses the anti-Staphylococcus aureus activity of neutrophils
Source: Protein Cell. 2023 Jul 31;15(3):223–9. doi: 10.1093/procel/pwad047 (PMC10903974; doi:10.1093/procel/pwad047)
Supplement: pwad047_suppl_Supplementary_Materials [file pwad047_suppl_supplementary_materials.pdf]

Supplemental figure legends

Figure S1. Validation of S100A9 His107 methylation site-specific antibody (H107me) and construction of *Mettl9*$^{-/-}$ (*Mettl9* knockout) mice

(A) *In vitro* methylation assays were performed using purified enzymatically active mouse METTL9 and the substrate GST-S100A9 WT or H107G mutant in the presence of [^{3}H]SAM, followed by autoradiography or Ponceau S staining. **(B)** *In vitro* methylation of GST-S100A9 purified by mouse METTL9 in the presence of SAM, followed by immunoblotting analysis with an S100A9 His107 methylation site-specific antibody (H107me). The total level of S100A9 was used as a loading control. **(C)** Scheme of *Mettl9*$^{-/-}$ mouse construction. **(D)** Genotyping of *Mettl9*$^{-/-}$ mice. **(E)** Validation of the efficiency of *Mettl9* knockout. *Mettl9* mRNA levels were determined in bone marrow cells isolated from WT and *Mettl9*$^{-/-}$ mice. **(F, G)** Representative and statistical flow cytometry data of the percentages of the indicated immune cell populations in bone marrow cells from WT or *Mettl9*$^{-/-}$ mice.

Figure S2. Construction of *Mettl9* FLAG knock-in mouse strains

(A) Scheme of *Mettl9* FLAG knock-in mouse construction. **(B)** Genotyping of *Mettl9* FLAG knock-in mice. **(C)** Immunoblotting analysis of whole extracts of different tissues isolated from *Mettl9* FLAG knock-in and *Mettl9*$^{-/-}$ mice probed with anti-FLAG antibody. PN, peritoneal neutrophils.

Figure S3. Analysis of the indicated cells in abscess tissues on the indicated days

(A-C) Representative and statistical flow cytometry data of the indicated cells in abscess tissues from WT mice at the indicated days after i.d. inoculation with 3×10^{8} *S. aureus,* as shown in **Fig 1D**. Error bars represent S.E.M.

Figure S4. Purification of methylated S100A8/A9 recombinant proteins

(A) The workflow of purification of methylated S100A9 or methylated S100A8/A9. **(B)** LC–MS/MS fragmentation spectra analysis of purified methylated S100A9. The monomethylated residues at His107 are colored red. **(C)** Immunoblotting analysis of the His107me level of S100A9 recombinant protein purified from *E. coli* cells after cotransforming the *S100a9* plasmid with or without *Mettl9* plasmids. **(D)** ITC binding curves for purified unmethylated and methylated S100A9 (S100A9me) binding to zinc. **(E)** Immunoblotting analysis of purified methylated S100A8/A9 probed with anti-His107(1-me) specific, anti-S100A8, and anti-S100A9 antibodies.

Figure S5. Analysis of the anti-*S. aureus* phenotypes upon deletion of *Mettl9*

(A) Representative flow cytometry plots and statistical analysis of the indicated cell populations within abscess tissues from WT or *Mettl9*$^{-/-}$ mice **(Fig. 2A**). Each dot represents one abscess tissue. n=10, Error bars represent S.E.M. **(B)** Flow cytometry analysis of ROS levels in primary neutrophils isolated from WT or *Mettl9*$^{-/-}$ mice in the presence of PMA at the indicated concentrations for 30 min. Each dot represents one mouse. n=3. **(C, D)** Flow cytometry analysis of NETs formation using SYTOX Green staining in primary neutrophils isolated from WT or *Mettl9*$^{-/-}$ mice in the absence ("Control") or presence of 50 nM PMA. Statistical plots of the percentages of SYTOX Green-positive cells are shown on the right. Each dot represents one mouse. n=4.

Figure S6. The correlation between *S. aureus* growth (OD_{600}) and lysate concentrations (µg/mL) at different TPEN concentrations

(A) Analysis of *S. aureus* growth in the presence of TPEN at the indicated concentrations in TSB medium. **(B-I)** Analysis of *S. aureus* growth in the presence of neutrophil lysate concentrations ranging from 0-1500 µg/mL and TPEN concentrations ranging from 0-200 µM in TSB medium. For all panels, error bars represent S.D.

Figure S7. Construction of the *S100a9*^{H107G} mouse strain

(A) Scheme of constructing the *S100a9*^{H107G} mouse. (**B**) Genotyping of *S100a9*^{H107G} mice. **(C)** Immunoblotting analysis of whole extracts of bone marrow cells isolated from WT and *S100a9*^{H107G} mice probed with anti-His107(1-me)-specific and anti-S100A9 antibodies.

Supplemental Figures

Figure S1.

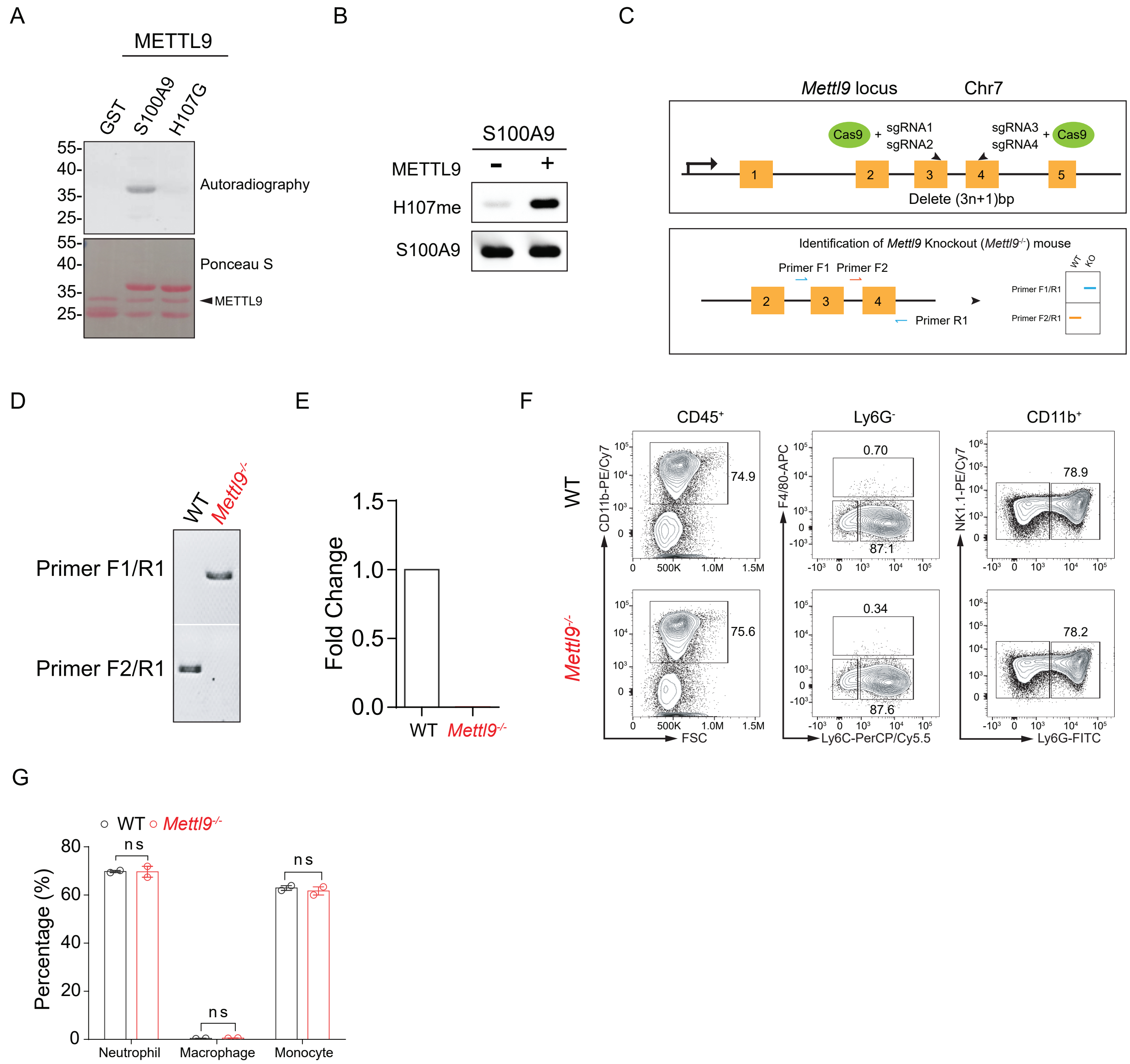

Figure S2

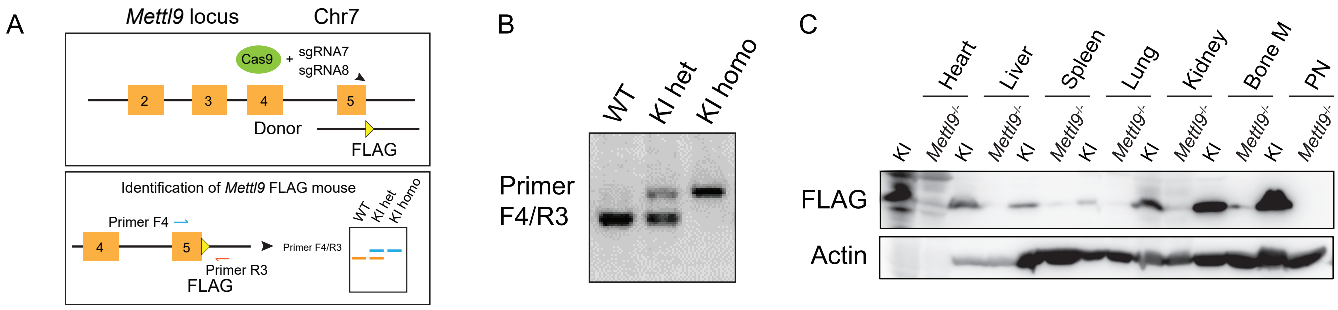

A
Mettl9 locus
Chr7
Cas9
+
sgRNA7
sgRNA8
2
3
4
5
Donor
FLAG
Identification of Mettl9 FLAG mouse
WT
KI het
KI homo
Primer F4
4
5
Primer F4/R3
Primer R3
FLAG
B
WT
KI het
KI homo
Primer F4/R3
C
Heart
Liver
Spleen
Lung
Kidney
Bone M
PN
KI
Mettl9-/-
FLAG
Actin

Figure S3.

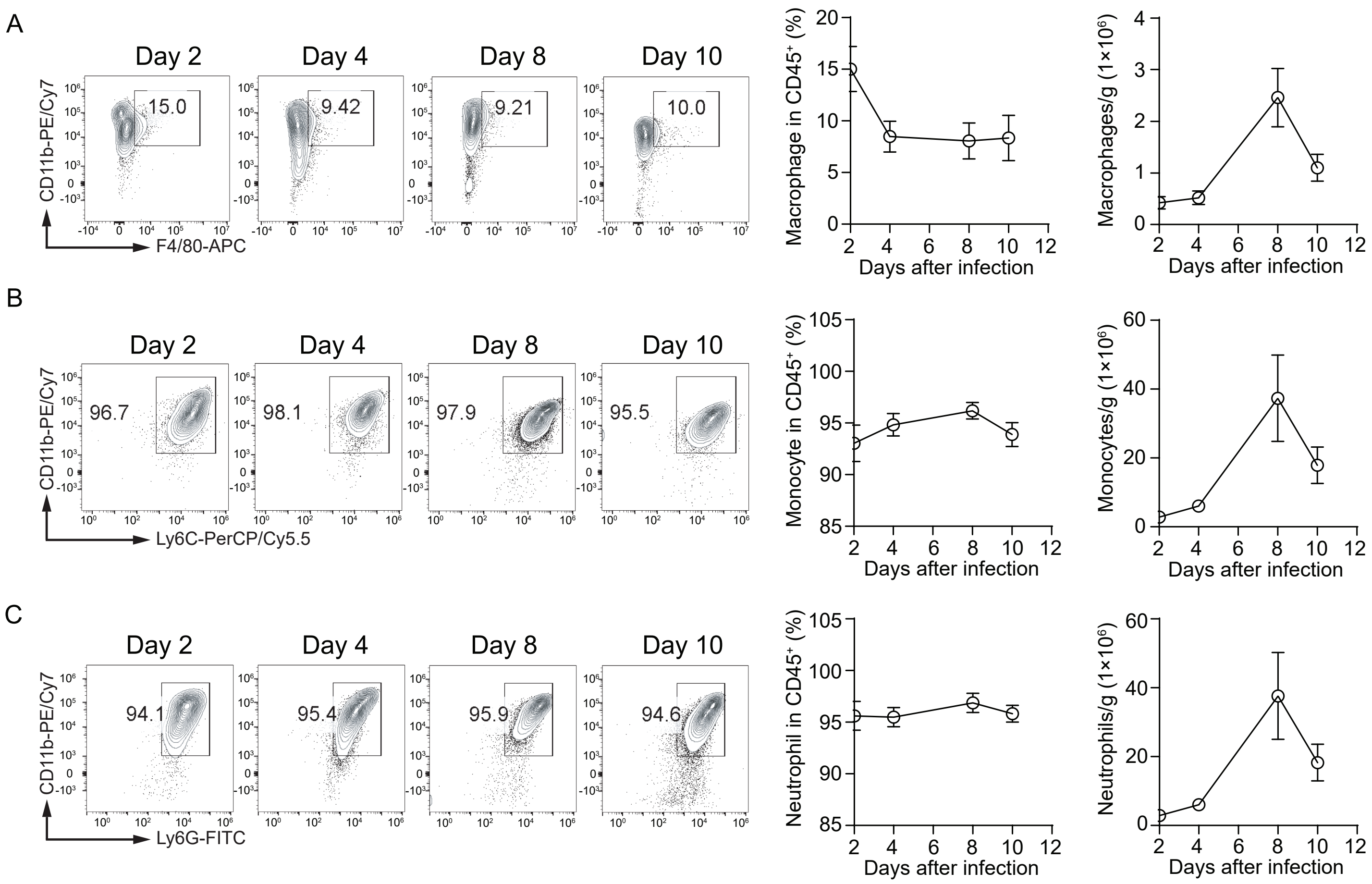

A
Day 2
Day 4
Day 8
Day 10
15.0
9.42
9.21
10.0
CD11b-PE/Cy7
F4/80-APC
Macrophage in CD45⁺ (%)
Macrophages/g (1×10⁶)
Days after infection
B
96.7
98.1
97.9
95.5
Ly6C-PerCP/Cy5.5
Monocyte in CD45⁺ (%)
Monocytes/g (1×10⁶)
C
94.1
95.4
95.9
94.6
Ly6G-FITC
Neutrophil in CD45⁺ (%)
Neutrophils/g (1×10⁶)

Figure S4.

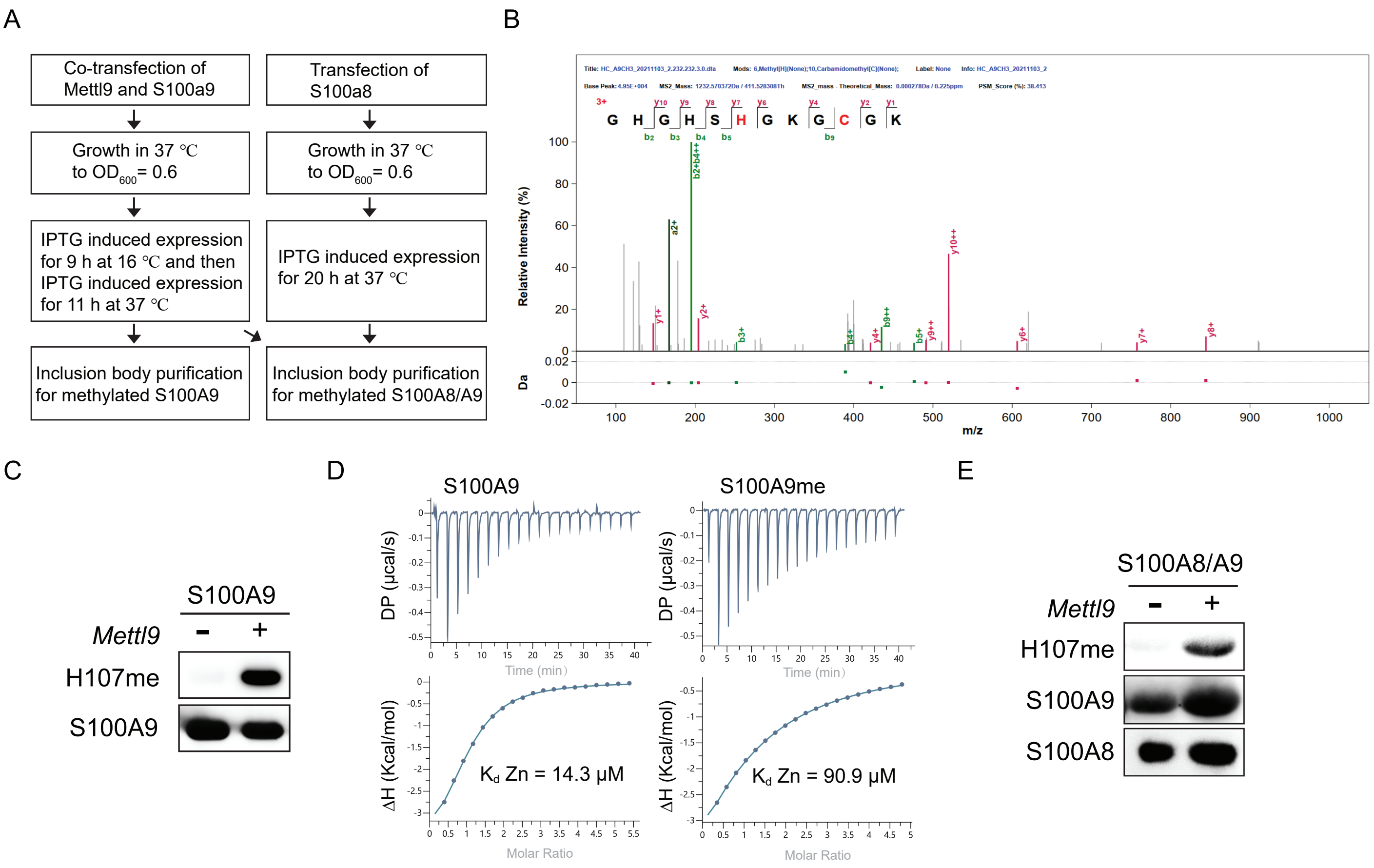

A
Co-transfection of Mettl9 and S100a9
Growth in 37 ℃ to OD_{600}= 0.6
IPTG induced expression for 9 h at 16 ℃ and then IPTG induced expression for 11 h at 37 ℃
Inclusion body purification for methylated S100A9
Transfection of S100a8
Growth in 37 ℃ to OD_{600}= 0.6
IPTG induced expression for 20 h at 37 ℃
Inclusion body purification for methylated S100A8/A9
B
G H G H S H G K G C G K
Relative Intensity (%)
Da
m/z
C
S100A9
Mettl9
H107me
S100A9
D
S100A9
S100A9me
DP (μcal/s)
Time (min)
ΔH (Kcal/mol)
Molar Ratio
K_d Zn = 14.3 μM
K_d Zn = 90.9 μM
E
S100A8/A9
Mettl9
H107me
S100A9
S100A8

Figure S5.

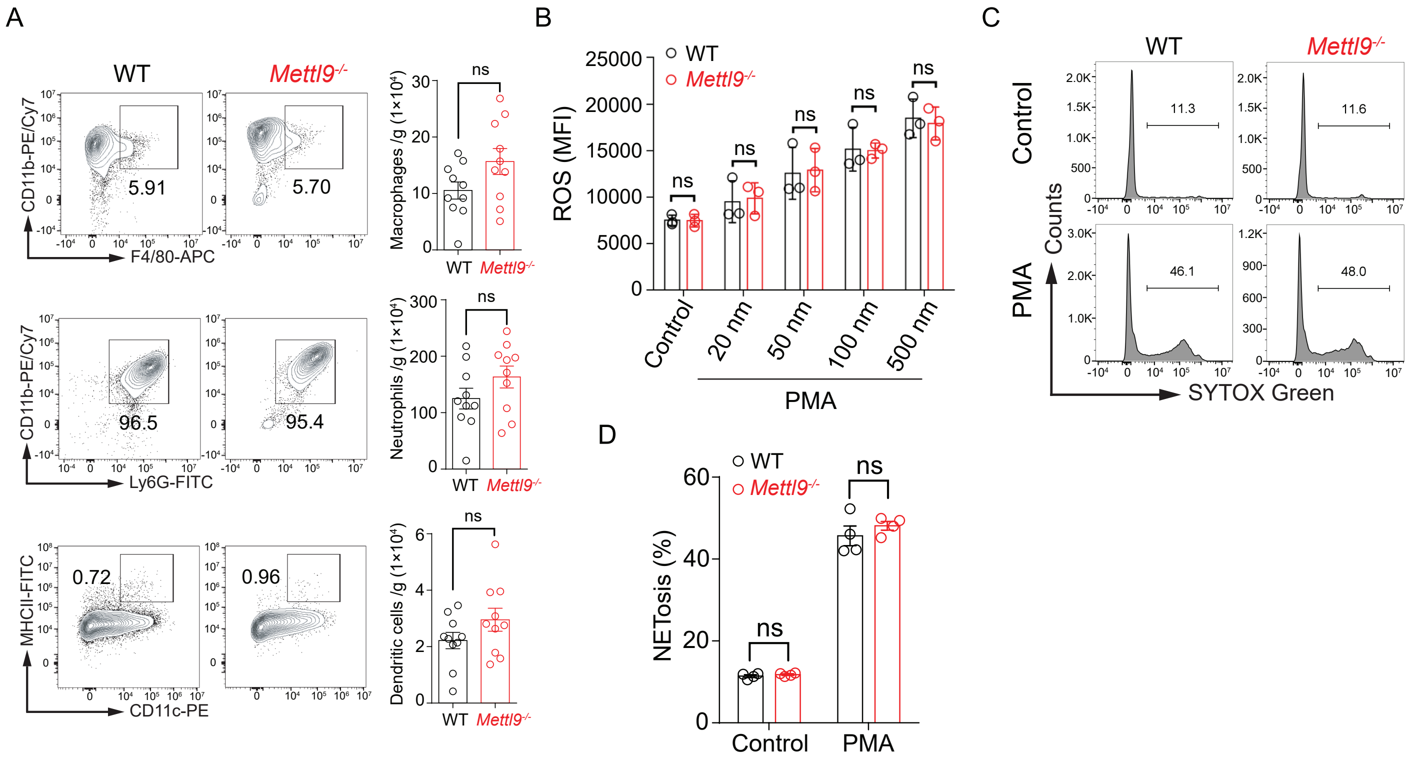

A
WT
Mettl9-/-
CD11b-PE/Cy7
F4/80-APC
5.91
5.70
Macrophages /g (1×10⁴)
ns
WT
Mettl9-/-
Ly6G-FITC
96.5
95.4
Neutrophils /g (1×10⁴)
MHCII-FITC
CD11c-PE
0.72
0.96
Dendritic cells /g (1×10⁴)
B
WT
Mettl9-/-
ROS (MFI)
Control
20 nm
50 nm
100 nm
500 nm
PMA
C
WT
Mettl9-/-
Control
PMA
Counts
11.3
11.6
46.1
48.0
SYTOX Green
D
WT
Mettl9-/-
NETosis (%)
Control
PMA

Figure S6.

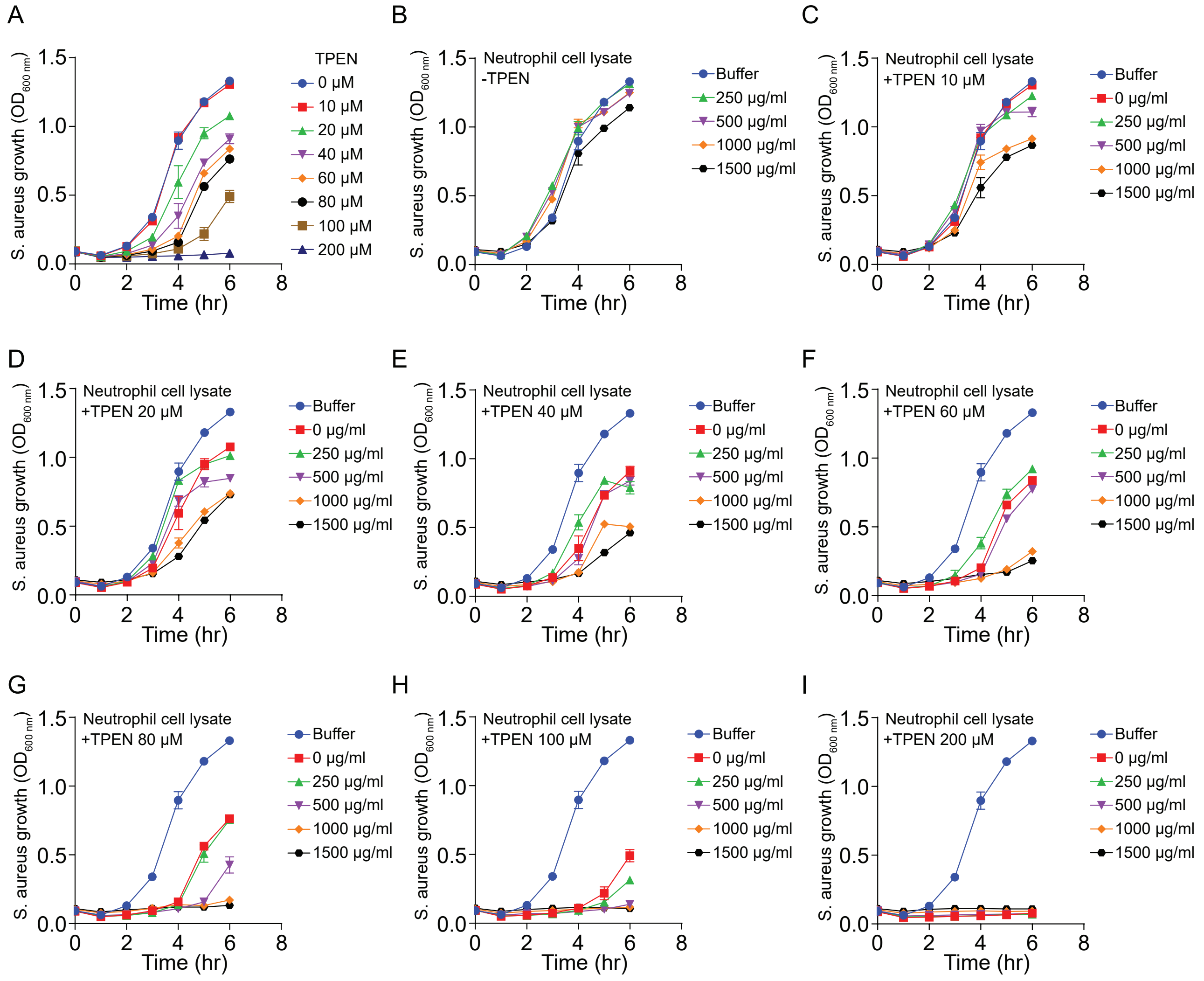

A
S. aureus growth ($OD_{600\ nm}$)
Time (hr)
TPEN
0 µM
10 µM
20 µM
40 µM
60 µM
80 µM
100 µM
200 µM
B
Neutrophil cell lysate
-TPEN
Buffer
250 µg/ml
500 µg/ml
1000 µg/ml
1500 µg/ml
C
Neutrophil cell lysate
+TPEN 10 µM
0 µg/ml
D
Neutrophil cell lysate
+TPEN 20 µM
E
Neutrophil cell lysate
+TPEN 40 µM
F
Neutrophil cell lysate
+TPEN 60 µM
G
Neutrophil cell lysate
+TPEN 80 µM
H
Neutrophil cell lysate
+TPEN 100 µM
I
Neutrophil cell lysate
+TPEN 200 µM

Figure S7.

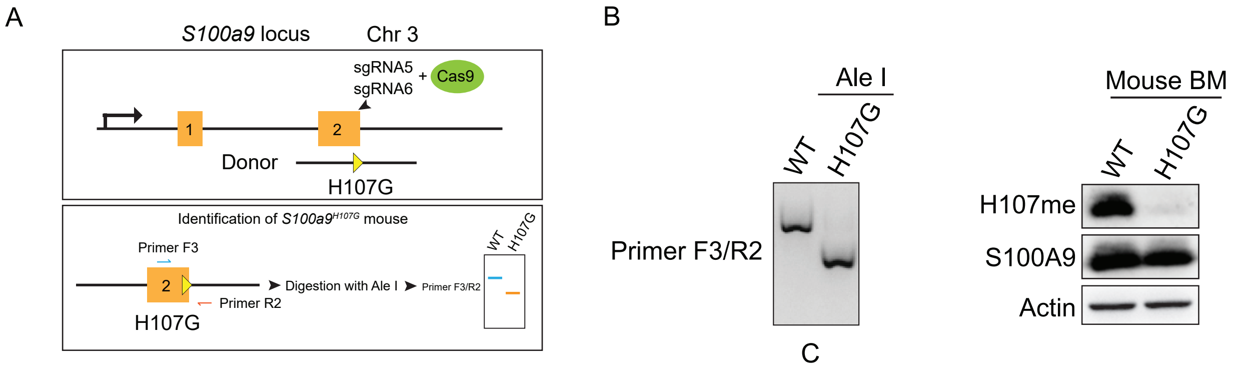
A
S100a9 locus
Chr 3
sgRNA5
sgRNA6
+
Cas9
1
2
Donor
H107G
Identification of S100a9H107G mouse
Primer F3
2
Primer R2
H107G
Digestion with Ale I
Primer F3/R2
WT
H107G
B
Ale I
WT
H107G
Primer F3/R2
C
Mouse BM
WT
H107G
H107me
S100A9
Actin

Supplemental Methods

Plasmids and bacterial strains

Mouse *S100a8*, *S100a9* and their mutants were cloned and inserted into the Nco I and Xho I sites of PET-28a. Mouse *Mettl9* was cloned and inserted into the EcoR I and Xho I sites of pGEX-6P-1. All the peptides were designed and inserted into pGEX-6P-1 by annealing. Site-specific mutations were performed using two reverse and complement primers containing the mutation codon.

S. aureus Newman was used for all relevant experiments in this study. One single clone was picked from tryptic soy broth (TSB medium, BD) agar plates, transferred to 3 mL fresh TSB medium, and cultured at 37°C and 220 rpm overnight. The next morning, 50 μL *S. aureus* culture was retransferred into 5 mL fresh TSB medium and cultured at 37°C and 220 rpm for 3~4 h to the logarithmic phase. The optical density (OD_{600}) of the *S. aureus* culture was determined by a spectrophotometer. To count the number of *S. aureus* in any given sample, the starting *S. aureus* culture was serially diluted at 1:10 (100 μL bacterial culture + 900 μL PBS) and vortexed gently to obtain a homogeneous mix. Ten microliters of bacterial dilution was spread on TSB agar plates in triplicate and cultured at 37°C overnight. The number of colonies was counted, and the CFU was calculated ((colony number × dilution factor)/coating volume). To calculate the cell density from OD_{600}, *S. aureus* culture was serially diluted, and the number of colonies formed on the dilution that gave the appropriate number was multiplied by the dilution factor to obtain the number of cells/mL in the original sample. These values were used to construct a calibration curve of OD vs. cells/mL.

Generation of anti-His107(1-me)-specific antibodies

For the generation of an anti-His107(1-me)-specific antibody (H107me), a peptide that corresponds to amino acids 100–113 of mouse S100A9 (PRGHGHSHGKGCGK) with His(1-me) at position 107 was synthesized and purified by high-performance liquid chromatography (99% purity). This peptide was conjugated to KLH and used as an antigen to immunize rabbits. Rabbit protocols, peptide conjugation, immunization and antiserum production were performed by HuaBio Company. Antiserum was negatively selected against an unmodified S100A9 peptide (amino acids 100–113). Final purification was performed with an immobilized antigenic peptide to select for His107(1-me)-specific antibodies.

Immunoblotting

To validate the specificity of the anti-His107(1-me)-specific antibody, S100A9 recombinant proteins were purified and used for *in vitro* methylation reactions. Then, the reactions were stopped by SDS–PAGE loading buffer (Tris 2.5 mM pH 6.8; 0.1% SDS; 0.005% bromophenol blue; 0.4% glycerol; 500 mM β-mercaptoethanol); the samples were heated at 100°C for 10 min and subjected to immunoblotting. For cell lysate, 4×10^6 bone marrow or peritoneal neutrophils were lysed on ice for 30 min in 80 μL RIPA buffer (50 mM Tris, 150 mM NaCl, 1% Triton X-100, 1% sodium deoxycholate, 0.1% SDS) with protease inhibitors (Complete Tablets EDTA-free, Roche, 04693132001) and PMSF (Beyotime, ST506) and were centrifuged at 12000 rpm and 4°C for 10 min; the supernatants were added to 20 μL SDS–PAGE loading buffer and heated at 100°C for 10 min, followed by immunoblotting. To detect secreted protein, the culture medium of PMA-stimulated neutrophils was collected and concentrated to 100 μL by centrifugation in Millipore® devices (cut-off 3 kDa). Then, 25 μL SDS–PAGE loading buffer was added to the concentrated medium and heated at 100°C for 10 min, followed by immunoblotting. For samples of tissues, 10 mg tissues were homogenized in 500 μL RIPA buffer, lysed on ice for 30 min with protease inhibitors and PMSF, and then centrifuged at 12000 rpm and 4°C for 10 min; the supernatants were added to 125 μL SDS–PAGE loading buffer and heated at 100°C for 10 min.

The target proteins were separated by 15% SDS–PAGE and transferred to polyvinylidene fluoride (PVDF) membranes (Millipore), followed by blocking with 5% nonfat-dried milk for 1 h at room temperature. PVDF membranes were probed with primary antibodies overnight and then probed with secondary antibodies conjugated to horseradish peroxidase (HRP) for 1 h at room temperature. The following antibodies were used: rabbit anti-His107(1-me) specific, rabbit anti-S100A9 (Invitrogen, PA5-82145), rabbit anti-S100A8 (Proteintech, 15792-1-AP), mouse anti-β-actin (Proteintech, 66009-1-Ig), mouse anti-FLAG (Abmart, M20008), HRP goat anti-mouse IgG (Beyotime, A2016), and HRP goat anti-rabbit IgG (Beyotime, A0208).

Mice

C57BL/6J wild-type (WT) mice were purchased from Beijing Vital River Laboratory Animal Technology. *Mettl9* knockout (KO, *Mettl9*$^{-/-}$) mice were generated by CRISPR–Cas9 technology as shown in Figure S1 (sg1: 5'-AAAGATGATGATCGGCCTCA-3'; sg2: 5'-GATGATGATGATCGGCCTCAGGG-3'; sg3: 5'-AGCCATGTGTAGTATCACCG-3'; sg4: 5'-TACAGCATTCTGTACGCCCC-3'). Genotyping of *Mettl9*$^{-/-}$ mice was performed using primers: Primer F1: 5'-GTGGTATGTGTGCAACAGAGAGAAA-3', Primer F2: 5'-AGCCTTGAGTCTCTGCCATTC-3' and Primer R1: 5'-CTGGCCTTGCATTCAATTCCC-

3', with amplicons of a 379-bp product from the WT allele and a 443-bp product from the targeted allele. *S100a9*^{H107G} mutant mice were generated as shown in Figure S6 (sg5: 5'-TTTTTGTAGGTCGTTGGCCC-3'; sg6: 5'-CTCAGAGAGCCCTCTGCCGT-3'). Donor DNA was produced with GGT (Gly) instead of CAT (His) at the His107 site, forming a new restriction enzyme AleI site for further genotyping. Genotyping of *S100a9*^{H107G} mutant mice was performed using the following primers: Primer F3: 5'-TCAGAGTTCTCTTCCTTAGCCCTA-3' and Primer R2: 5'-CTGTTTATCACTCAGAGCCTGGGA-3'. Amplicons were digested with the restriction enzyme AleI at 37°C for 2 h; the targeted allele was cut into two identical small bands. *Mettl9* FLAG knock-in (KI) mice were generated as shown in Figure S2 (sg7: 5'-CTCAGACCAGTATAAACACG-3'; sg8: 5'- AGACCAGTATAAACACGTGG -3'). Donor DNA was produced with a FLAG sequence (5'-GACTACAAGGACCACGACGGTGACTACAAGGACCACGACATCGACTACAAGGACGACGACGACAAG-3') inserted just before the stop codon of *Mettl9*. Genotyping of *Mettl9* FLAG KI mice was performed using the following primers: Primer F4: 5'-GAAGGCGACATGTACAATGACTAC-3' and Primer R3: 5'-CTCACATCACTGGATGCATCTC-3', with amplicons of a 106-bp product from the WT allele and a 172-bp product from the targeted allele. All mice had a C57BL/6J background and were housed in specific pathogen-free conditions. All mice were maintained under a 12-h light-dark cycle at 23°C and had free access to water and standard rodent diet. All mice were used at 8 to 12 weeks of age. All experimental procedures involving mice were approved by the Ethics Committee of USTC (reference: USTCACUC192401055).

RT–qPCR

Total RNA was extracted and purified from cells using TRIzol reagent (TIANGEN) and reverse-transcribed to cDNA using HiScriptIII RT-SuperMix for qPCR (Vazyme, R323) according to the manufacturer's instructions. qPCR was performed using ChamQ SYBR qPCR Master Mix (Vazyme, R311) on a CFX384 Real-Time System (Bio-Rad), and the primer pairs used for the target genes were as follows: *Mettl9*-F: ACGGGTTGCTAGGAAGAGG; *Metll9*-R: ACTGGAACCCTGTATTCTGCC. *Rpl13a*-F: CGAGGTTGGCTGGAAGTACC; *Rpl13a*-R: CTTCTCGGCCTGTTTCCGTAG

***In vitro* methyltransferase assays**

In vitro methyltransferase assays were performed using 30 μL reaction buffer (50 mM Tris-HCl pH 7.8, 50 mM KCl, 5 mM $MgCl_2$) with 1 μL [^{3}H]SAM ([^{3}H]S-(5′-adenosyl)-L-methionine) (Perkin Elmer, specific activity = 55-85 Ci/mMole, 0.55 μCi/μL) or 1 mM unlabelled SAM (S-(5′-adenosyl)-L-methionine) (Sigma–Aldrich). For the methylation of recombinant proteins, reactions were performed using 2 μg substrates and 2 μg METTL9, incubated at 37°C for 1 h, and stopped by the addition of SDS–PAGE loading buffer. Proteins were separated by SDS–PAGE. For autoradiography analysis, the proteins were transferred to PVDF membranes, stained with Ponceau S and exposed to XBT X-ray film (Carestream). The exposure time ranged from one to four weeks, depending on the methylation efficiency. For western blotting, the proteins were transferred to PVDF membranes and probed with the indicated antibodies.

Purification of S100A8/A9 recombinant proteins

Purification of S100A8/A9 wild-type or H107G recombinant proteins was performed using a modified version of a previous protocol(1). Briefly, the S100A8 or S100A9 plasmid was transformed into BL21(DE3) chemically competent E. coli cells separately. The transformed E. coli cells were grown, and protein expression was induced. E. coli cells were collected and sonicated on ice. The lysate was centrifuged to gather the inclusion bodies. The pellet of inclusion bodies was washed and solubilized in refolding buffer. At this stage, the denatured inclusion bodies of wild-type or mutant S100A8 and S100A9 were combined and loaded onto a hydroxyapatite column. Next, S100A8/A9 wild-type or mutant recombinant protein-containing fractions were dialyzed and then loaded onto and eluted from a Source Q column. Fractions containing S100A8/A9 wild-type or mutant recombinant proteins were analysed via SDS–PAGE.

Purification of methylated S100A8/A9 recombinant proteins was performed by transforming *Mettl9* and *S100a9* wild-type plasmids together into BL21(DE3) chemically competent *E. coli* cells using standard protocols. The *S100a8 plasmid was transformed separately*. The *E. coli* cells were grown overnight at 37°C on LB/agar plates supplemented with 100 μg/mL ampicillin and kanamycin to screen colonies coexpressing METTL9 and S100A9. The screened colony was grown in LB medium at 37°C until the optical density (OD_{600}) reached ~0.6. Protein expression was induced with 0.5 mM IPTG for 9 h at 16°C and then for 11 h at 37°C. The remaining steps for protein purification were the same as above. Note that the denatured inclusion bodies of S100A8 and S100A9 were combined for

renaturation. For further analysis of protein methylation, the samples were detected by anti-His107(1-me)-specific antibody or sent for MS detection.

The processed MS data were analysed with ProteinDiscovery (version 2.2, Thermo Fisher Scientific) against the UniProt mouse filtered organism database or in-house database, including the amino acid sequences of synthetic peptides, using the following parameters: enzyme = trypsin; maximum missed cleavages = 1; variable modifications = acetyl (protein N-term), oxidation (M), methyl (H), methyl (K), methyl (R), and propionamide (C); product mass tolerance = ±10 ppm; product mass tolerance = ±0.02 Da (LIFT mode); instrument type = Orbitrap.

Antibacterial activity assays of S100A8/A9 recombinant proteins.

The antibacterial activity assays of the S100A8/A9 recombinant protein were performed as previously described(2). Briefly, *S. aureus* was grown overnight at 37°C in 5 mL of TSB medium. The next morning, the *S. aureus* was back-diluted 1:50 into 5 mL fresh TSB medium in a 15 mL conical tube and grown for 1 h at 37°C on a roller drum. Following this incubation, exponential phase *S. aureus* was diluted 1:100 into 96-well round-bottom plates, which already contained 38% (vol/vol) TSB and 62% (vol/vol) S100A8/A9 buffer (100 mM NaCl, 3 mM CaCl2 10 mM β-mercaptoethanol, 20 mM Tris, pH 7.5) with various concentrations of unmethylated, methylated S100A8/A9, or S100A8/A9 H107G recombinant proteins. Bacteria were grown at 37°C with shaking at 220 rpm, and the optical density (OD_{600}) was measured to monitor growth.

Isothermal titration calorimetry (ITC)

The affinity of proteins for zinc ions was determined using a MicroCal iTC200 system. Proteins and metal solutions were prepared in 20 mM Tris at pH 7.5, 100 mM NaCl and 5 mM β-mercaptoethanol. The S100A9 or S100A9me protein solution (at concentrations ranging from 10 µM to 40 µM) was loaded in the calorimetric cell. $ZnSO_4$ (at concentrations of 1 mM) was titrated in the protein sample at 25°C for 20 injections of 2 µL each. The dissociation constant (K_d), enthalpy of binding (ΔH), and stoichiometry (N) were obtained after fitting the integrated and normalized data to a single-site binding model by MicroCal PEAQ-ITC Analysis software (Malvern Panalytical). All experiments were performed at least in duplicate to ensure the reproducibility of the data. Binding isotherms were plotted, analysed and fitted based on a one-site binding model by MicroCal PEAQ-ITC Analysis software (Malvern Panalytical) after subtraction of the respective controls. K_ds were averages of individual K_d calculations from

duplicated experiments.

Isolation of bone marrow neutrophils

For isolation of bone marrow leukocytes, bone marrow was crushed by using a mortar and pestle; the remaining aggregates and debris were removed by passing the cell suspension through a 70 μm mesh nylon strainer, and leukocytes were obtained after lysing erythrocytes. The cells were centrifuged at 300 × g for 10 min and suspended at 1×10^8 nucleated cells/mL in MACS buffer (PBS containing 2% fetal bovine serum (FBS) and 1 mM EDTA). For the isolation of neutrophils, cells were purified from bone marrow leukocytes through magnetic activated cell sorting (MACS) using a negative selection cell isolation kit (EasySep™ Mouse Neutrophil Enrichment Kit, STEMCELL).

Analysis of neutrophil NETs and ROS

Bone marrow-derived neutrophils were isolated using an immunomagnetic negative selection cell isolation kit (EasySep™ Mouse Neutrophil Enrichment Kit, STEMCELL). A total of 1×10^6 neutrophils were resuspended in 500 μL of 1640 medium and added to a 1.5 mL microcentrifuge tube. Then, 5 μL of 1:250 diluted SYTOX Green (Thermo Fisher) was added to the cell suspension. Next, 500 μL of the DMSO- or PMA-supplemented media was added to the microcentrifuge tubes, with a final concentration of 30 nM. The samples were then added to a 96-well cell culture plate in quadruplicate and incubated for 2 h at 37°C. After incubation, SYTOX $Green^+$ cells were measured using a flow cytometer.

The total intracellular ROS level was determined using dichlorodihydrofluorescein diacetate (DCFH-DA, Beyotime). Briefly, 1×10^6 neutrophils were incubated with 10 μM DCFH-DA in 1 mL serum-free 1640 medium at 37°C for 20 min. Cells were then washed three times with 1640 medium (serum-free) and treated with DMSO or PMA (20-500 nM) in 1 mL serum-free 1640 medium for 30 min at 37°C. After washing with PBS, the relative ROS level of the cells was detected by flow cytometry at a 488 nm excitation wavelength and 520 nm emission wavelength.

Antibacterial activity assays of neutrophil lysates

Mice were injected intraperitoneally (i.p.) with 4% thioglycolate at 18 h and 3 h prior to euthanasia. To collect the peritoneal exudate cells (PECs), 5 mL PBS was injected into the peritoneal cavity, and the injected wash was subsequently withdrawn while gently massaging the peritoneal wall. The PECs were subsequently resuspended in MACS buffer for flow cytometry analyses, MACS isolation of neutrophils, or bacterial killing assays as described

below. For lysate killing, *S. aureus* Newman were grown overnight, reinoculated 1:50 in TSB medium at 37°C, 220 rpm for 1 h and then diluted 1:100 for the following experiments; 3×10^7 neutrophil cells were lysed in S100A8/A9 buffer (100 mM NaCl, 3 mM $CaCl_2$, 10 mM β-mercaptoethanol, 20 mM Tris, pH 7.5); ultrasound was performed at 40% power for 5 min. *S. aureus* culture was mixed with neutrophil lysates and TPEN (Sigma–Aldrich) in TSB medium, grown at 37°C with shaking at 220 rpm in a 96-well microtiter plate, and the OD_{600} value was monitored over time. To optimize the antibacterial effects of cell lysates in inhibiting *S. aureus* growth, lysate concentrations (µg/mL) ranging from 0-1500 µg/mL (approximately 100-600 µg/mL S100A8/A9 in lysates) and TPEN concentrations ranging from 0-100 µM in TSB medium were used to establish the correlation between *S. aureus* growth (OD_{600}) and lysate concentration (µg/mL) at a given TPEN concentration. To assess the antibacterial activity of WT and *Mettl9*$^{-/-}$ cell neutrophil lysates, *S. aureus* was incubated with 350 µg/mL lysate and 100 µM TPEN in TSB medium in a 96-well microtiter plate, and *S. aureus* growth was monitored by measuring the increase in OD_{600} over time. To assess the Zn^{2+}-rescued antimicrobial properties, excessive Zn^{2+} was added to the above system at a final concentration of 500 mM. To assess the antibacterial activity of *S100a9*^{H107G} and *Mettl9*$^{-/-}$ *S100a9*^{H107G} cell neutrophil lysates, *S. aureus* was incubated with 500 µg/mL lysate and 40 µM TPEN in TSB medium in a 96-well microtiter plate, and *S. aureus* growth was monitored by measuring the increase in OD_{600} over time.

Stimulation of primary neutrophils

Mettl9 FLAG KI mice were injected intraperitoneally (i.p.) with 4% thioglycolate at 18 h and 3 h prior to euthanasia. Peritoneal cells were collected as described previously. A total of 4×10^6 cells were stimulated with 1 µg/mL LPS or 4×10^7 *S. aureus* Newman for 0 h, 1 h, 3 h, or 6 h in 6-well plates. At each time point, the cells were lysed on ice for 30 min in 64 µL RIPA buffer with protease inhibitors and PMSF and then centrifuged at 12000 rpm and 4°C for 10 min. The supernatants were collected, and 16 µL SDS–PAGE loading buffer was added and heated at 100°C for 10 min, followed by immunoblotting.

Model of *S. aureus* skin infection

Six- to eight-week-old sex-matched WT C57BL/6J, *Mettl9*$^{-/-}$, *S100a9*^{H107G} or *Mettl9*$^{-/-}$ *S100a9*^{H107G} mice were shaved on the flank and allowed to rest for two days. *S. aureus* Newman was grown in TSB medium, washed three times in sterile PBS, and then inoculated subcutaneously into both sides of the flank with 3×10^8 *S. aureus* in 100 µL PBS. For the

recombinant protein-mediated *in vivo* killing assay, WT mice were inoculated subcutaneously into both sides of the flank with 2×10^8 *S. aureus* in 100 µL PBS with 30 µg protein. Lesion areas were measured daily, and mice were euthanized 3-5 days after infection. One-third of the abscess tissue was fixed in 4% paraformaldehyde (PFA) for histology. To determine the bacterial numbers in the abscess tissue, the other one-third of abscess tissue was weighed and homogenized in cold PBS, and the tissue homogenate was serially diluted and plated onto TSB agar plates. The number of CFUs was determined after 24 h of incubation at 37°C. To isolate the abscess-infiltrating leukocytes, the remaining abscess tissue was weighed, cut into pieces and digested in RPMI 1640 medium with 1 mg/mL collagenase IV and 20 µg/mL DNase I for 40 min at 37°C. Suspensions were filtered through sieves, and leukocytes were obtained by centrifugation. The single-cell suspension was incubated with anti-CD16/32 mAbs to block Fc receptors, followed by staining with fluorescence antibodies against surface molecules for 30 min at 4°C. The following antibodies were purchased from BioLegend: APC/Cyanine7 anti-mouse CD45.2 (Clone 104), PE anti-mouse CD11c (Clone N418), PE/Cyanine7 anti-mouse/human CD11b (Clone M1/70), APC anti-mouse F4/80 (Clone BM8), FITC anti-mouse Ly-6G (Clone 1A8), PerCP/Cyanine5.5 anti-mouse Ly-6C (Clone HK1.4), PE/Cyanine7 anti-mouse NK-1.1 (Clone PK136), and FITC anti-mouse I-A/I-E (Clone M5/114.15.2). All data were collected on a BECKMAN COULTER CytoFLEX S flow cytometer and analysed with FlowJo software (Tree Star).

Statistical Analysis

Statistical analyses were performed using Prism 8.0 (GraphPad version 8). Multiple comparisons were analysed using one-way ANOVA. Normal distributions were analysed by Student's t test. For time-course analysis of OD_{600}, two-way ANOVA was used. Differences at $p < 0.05$ were considered significant. Statistical parameters are represented in the Figure Legend of each figure. $P < 0.05$ was considered significant. *$p < 0.05$, **$p < 0.01$, ***$p < 0.001$, and ****$p < 0.0001$.

References

1. Hunter MJ & Chazin WJ (1998) High level expression and dimer characterization of the S100 EF-hand proteins, migration inhibitory factor-related proteins 8 and 14. *The Journal of biological chemistry* 273(20):12427-12435.
2. Kehl-Fie TE*, et al.* (2011) Nutrient metal sequestration by calprotectin inhibits bacterial superoxide defense, enhancing neutrophil killing of Staphylococcus aureus. *Cell Host Microbe* 10(2):158-164.
